# Intramural haematoma of the thoracic aorta: who's to be alerted the cardiologist or the cardiac surgeon?

**DOI:** 10.1186/1749-8090-4-54

**Published:** 2009-10-01

**Authors:** Nikolaos G Baikoussis, Efstratios E Apostolakis, Stavros N Siminelakis, Georgios S Papadopoulos, John Goudevenos

**Affiliations:** 1 Cardio-thoracic Surgery Department, University Hospital of Patras, School of Medicine, Patras, Greece; 2 Cardiac Surgery Department, University Hospital of Ioannina, School of Medicine, Ioannina, Greece; 3 Department of Clinical Anaesthesiology and Intensive Postoperative Care Unit, University Hospital of Ioannina, School of Medicine, Ioannina, Greece; 4 Department of Cardiology, University Hospital of Ioannina, School of Medicine, Ioannina, Greece

## Abstract

This review article is written so as to present the pathophysiology, the symptomatology and the ways of diagnosis and treatment of a rather rare aortic disease called Intra-Mural Haematoma (IMH). Intramural haematoma is a quite uncommon but potentially lethal aortic disease that can strike as a primary occurrence in hypertensive and atherosclerotic patients to whom there is spontaneous bleeding from vasa vasorum into the aortic wall (media) or less frequently, as the evolution of a penetrating atherosclerotic ulcer (PAU). IMH displays a typical of dissection progress, and could be considered as a precursor of classic aortic dissection. IMH enfeebles the aortic wall and may progress to either outward rupture of the aorta or inward disruption of the intima layer, which ultimately results in aortic dissection. Chest and back acute penetrating pain is the most commonly noticed symptom at patients with IMH. Apart from a transesophageal echocardiography (TEE), a tomographic imaging such as a chest computed tomography (CT), a magnetic resonance (MRI) and most lately a multy detector computed tomography (MDCT) can ensure a quick and accurate diagnosis of IMH. Similar to type A and B aortic dissection, surgery is indicated at patients with type-A IMH, as well as at patients with a persistent and/or recurrent pain. For any other patient (with type-B IMH without an incessant pain and/or without complications), medical treatment is suggested, as applied in the case of aortic dissection. The outcome of IMH in ascending aorta (type A) appears favourable after immediate (emergent or urgent) surgical intervention, but according to international bibliography patients with IMH of the descending aorta (type B) show similar mortality rates to those being subjected to conservative medical or surgical treatment. Endovascular surgery and stent-graft placement is currently indicated in type B IMH.

## Introduction

Intramural haematoma (IMH) belongs to "acute aortic syndrome" followed by penetrating atherosclerotic ulcer (PAU) and the classical acute aortic dissection. It occurs as a bleeding into the aortic wall (media) without initial rupture of the intima and the classic flap formation. Despite IMH of the thoracic aorta being a disease of the aorta, its optimal initial treatment still remains a hot debatable issue. Aortic intramural haematoma can firstly appear to hypertensive and atherosclerotic patients who suffer an impulsive haemorrhage from vasa vasorum rupture into the media either spontaneously, or less commonly, as a result of PAU. Rarely does a thoracic trauma lead to IMH [1,2]. According to records [3], the IMH can successfully be cured; lead to aortic dissection or in aortic rupture. The initial haematoma of the aortic wall may be augmented and further affect the medial layer of the aorta [3], similarly to the aortic dissection. Consequently, IMH weakens the aorta and may progress to either outward rupture of the aortic wall or inward disruption of the intima, which leads to aortic dissection [4-6]. Similarly to aortic dissection, IMH is divided in proximal (type A) and distal, without the ascending aorta being involved (type B). According to recommendations of the Task Force on aortic dissection, European Society of Cardiology, there are two types of IMH: Type I shows a smooth inner aortic lumen, the diameter usually being less than 3.5 cm, and the wall thickness is bigger than 0.5 cm [3,7]. The Type II IMH occurs in aortic atherosclerosis. A rough inner aortic surface with severe aortic sclerosis is frequently noticed. The aorta is dilated to more than 3.5 cm and calcification is usually found. Mean wall thickness is 1.3 cm ranging from 0.6 to 4 cm [3,7]. As far as the clinical impact of intramural haematoma is concerned, it has been documented that the cases of type A haematomas tend to have a high frequency of complications (dissection or rupture) even death, and therefore should be surgically treated in emergent or urgent setting [4,8]. Contrary to the above, type B-distal, (descending aorta), IMH uncommonly progress to complications and is frequently completely resolved without any intervention [8]. In the era of endovascular surgery the stent-graft placement in the descending aorta has an indication.

### Pathogenesis and pathophysiology of the IMH

The common risk factors for cardiovascular diseases are to be held responsible in the pathogenesis of the IMH. Special circumstances, such as pregnancy as well as some congenital disorders should be taken into account. Arterial hypertension is the most frequent predisposing factor for IMH, present in 84% of the patient cohort and similarly to the 67% incidence, reported in a post mortem study of 161 cases of dissection [1,9]. Nevertheless, as in the case of aortic dissection, the initiating event of acute IMH remains unknown. Nutrient vases called Vasa vasorum are present in most arteries, including the aorta and coronary arteries, carotids, and femoral arteries [10]. Pathological neovascularization of the vessel wall is a consistent feature in the formation of atherosclerotic plaque and development of the disease [11,12]. Additionally, microvessels are increased in coronary lesions from patients with acute myocardial infarction, suggesting a potential role of microvessels in plaque rupture and instability [13]. Furthermore, microvessels play a role in plaque haemorrhage associated with the development of symptoms in cerebrovascular disease according to some reports [14,15]. Gore [16] suggested that spontaneous rupture of aortic vasa vasorum may initiate aortic wall disintegration, eventually leading to dissection. Moreover, rupture of the nutrient vasa vasorum of the media layer may cause haematoma without a tear [3,14]. Other authors have proposed intimal "fracture" of an atherosclerotic plaque as the primary event, which then allows propagation of blood into the aortic media causing intramural haematoma. Moreover, discrete penetrating atheromatous ulcers have also been thought as a prerequisite for intramural bleeding [17]. In such a chronic setting, however, the haematoma is confined to the area nearest to the atherosclerotic ulcer. Although some uncertainty exists concerning how to distinguish IMH from limited aortic dissection with a thrombosed false lumen, IMH pathology has been identified as the very early stages of dissection with an impending risk of rupture [18,19]. What is more, patients with intramural haematoma are also typically older than those with classic dissection, supporting the opinion that degenerative changes in the media play a key role in the IMH formation [5]. There are some both acquired and genetic conditions leading to the breakdown in the integrity of the intima, which weaken the media layers of the aorta, and lead to a higher aortic wall stress [6,20]. As a final result, these factors may induce an aortic dilation, aneurysm formation, intramural beeding, acute or chronic aortic dissection, or aortic rupture. Furthermore, the extracellular matrix may be subjected to degradation, apoptosis, and elastolysis, frequently at the limits of the atherosclerotic plaques [1,2,21]. However, a series of congenital abnormalities such as Marfan's syndrome, Ehlers-Danlos syndrome [3] annuloaortic ectasia, bicuspid aortic valve, and familial aortic dissection are the prime suspects, predisposing acute aortic syndromes [3,7,22]. Stefanadis's study carried out with dogs experimented, revealed that aortic wall distensibility decreased significantly to those with removed vasa vasorum of the aortic wall [20]. Atherosclerosis leads to the thickening of the intima layer of the vases. Thickness of the intima increases the distance between the endothelial layer and the media, compromising the nutrient and oxygen supply while adventitial fibrosis may obstruct small intramural vasa vasorum. Reduced nutritional supply of the media results in media thinning following a necrosis due to the necrosis of the smooth muscle cells. [3] Advanced imaging technology (MDCT) has defined precursors or "variants" to frank aortic dissection such as IMH, PAU, and localized intimal tears [3-8,23,24]. Ramona Scotland et al characterize endothelin-1 (ET-1)-mediated contraction of vasa vasorum and investigate whether threshold concentrations of ET-1 alter any sensitivity to constrictors [25]. Circulating plasma levels of ET-1 are elevated in several disease including atherosclerosis, hypertension, congestive heart failure asthma and diabetes. In our opinion, after the atheroschlerotic plaque destabilization, a spontaneous plaque rupture follows. As we know, vassels (vasa vasorum), are also present in the atheroschlerotic plaque matrix. Is it the plaque rupture that provokes immediately vasa vasorum rupture and intramural haematoma? The presence of several layers of smooth muscle implies that the vessels of the vasa vasorum actively regulate their own tone rather than serving as a passive channel for the blood flow. Many studies have been conducted with dogs in vivo supporting this hypothesis investigating vasa vasorum reactivity to vasoactive agents. Heistad suggested that the diameter of the vasa vasorum of canine thoracic aorta increases in response to intravenous infusion of adenosine [26].

## Diagnosis

**Figure 1:**
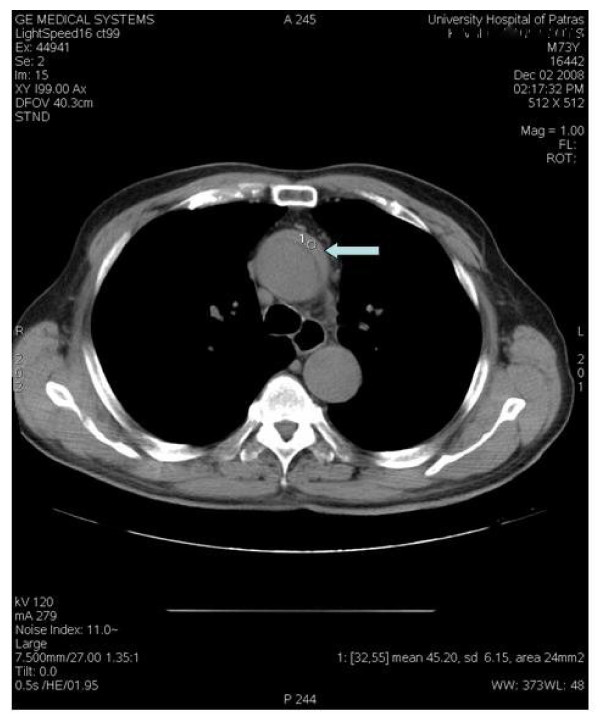
**Contrast-enhanced CT reveals an intramural haematoma (IMH) of the ascending aorta located mainly in the anterolateral wall (arrow)**. The haematoma is appeared as a thickening of the aortic wall.

**Figure 2:**
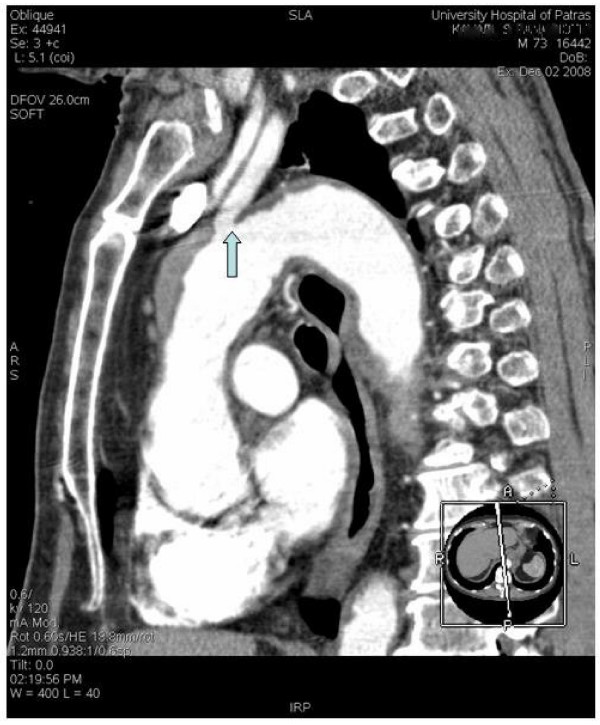
**Oblique reformation image of a contrast-enhanced CT scan of the same patient**. The IMH is appeared as a thickening of the aortic wall extended in the aortic arch compressing the origin of the brachiocephalic artery (arrow).

The IMH is diagnosed in the same way as with acute aortic dissection. In reality, the clinical symptomatology of IMH may be virtually indistinguishable from that of acute dissection. Chest and back pain is reportedly as the most frequent clinical manifestation in patients with IMH [27]. Chest pain is more common with ascending (proximal-type A) IMH; upper or lower back pain is more common with descending (distal-type B) lesions [28]. Patiens with acute aortic dissection may suffer renal and hepatic ischemia due to malperfusion; malperfusion and pulse deficit are decidedly rare in IMH because of its local limitation [5,27]. During physical examination some suspicion of a serious aortic disease should arise. As in aortic dissection, a widening of the mediastinum or the aortic shadow and pleural effusion may be illustrated in the x-ray. [29-31]. Acute myocardial infarction can resemble the acute aortic syndrome and can be dangerous if not correctly diagnosed. The ECG must be applied at all patients because it helps distinguish acute myocardial infarction, for which thrombolytic therapy may be life saving, from aortic dissection or acute aortic syndrome, for which thrombolytic therapy may be detrimental [3]. Consequently, the trans-thoracic ultrasuonography is useful but not diagnostic but the trans-esophageal echocardiography will demonstrate localized thickening of the aorta with a "thrombuslike appearance" characterized by echo-lucent areas, and compression of the true lumen [32]. Intimal displacement of calcium may be evident when using this technology, just as it may happen with other imaging modalities. Sensitivity of transesophageal echocardiography has been reported to be as high as 100% with a specificity of 91%, although this as it is known, will be operator dependent [33]. In other studies sensitivity and specificity of trans-thoracic echocardiography range from 77% to 80% and 93% to 96%, respectively, for the involvement of the ascending aorta [3]. According to the Task Force on aortic dissection, European Society of Cardiology, Echo-free spaces (seen echocardiographically) as a sign of intramural haematoma are found in only one third of the patients. The mean longitudinal extent of the haematoma is 11 cm and the echo free spaces show no signs of flow [7]. In patients with a Type II IMH echo free spaces are found in 70%. The longitudinal extension has a range similar to type I haematoma, usually about 11 cm [3,7]. The capability of this diagnostic tool of finding an intimal flap and thereby distinguish IMH from dissection with thrombosis is limited [34]. In this way, a CTA is necessary for the diagnosis and the treatment. We are to present two interesting images of one of our patients, treated by the authors (figure 1, 2); in figure 1 an IMH is shown in ascending aorta (type A). This patient was treated surgically in emergency setting. In figure 2 the same patient with IMH placed in the aortic arch. Our patient underwent a Bentall procedure with hemiarch replacement through axillary artery cannulation [35]. In this way we were able to operate without any cerebral perfusion compromise. In a study of Nienaber et al the sensitivity of the thorax CT was nearly 100% [8]. According to studies, the majority (50% to 85%) is located in the descending aorta (type B) and are usually associated with hypertension [34,35]. In the case of IMH no dissection flap is present because the integrity of intima layer of the aortic wall is unexceptionable [6,8]. Recently [36], the multi-detector computed tomography (MDCT) has an important role in the diagnosis of the IMH. According to this report the accuracy provided, a chronic from an acute clot into the aortic wall is distinguished. Magnetic resonance imaging (MRI) may be superior to computed tomography in differentiating IMH from atherosclerotic plaque [37]. This is crucial, because the two findings have a completely different prognosis and the ways of treatment are different. MRI will demonstrate the thickening of the wall, with hyperintense foci indicative of bleeding on T1-weighted images, although the signal intensity characteristics depend partly on the age of the haematoma [32,38]. Cost and availability are of course two deterrents in MRI application; provincial Hospitals usually have neither the instrumentation nor the technology or equipment needed for MRI. Regardless of the technology employed, the extent and thickness of the IMH is important in order to compare with subsequent studies. Nienaber CA and colleagues demonstrated in 1995 that while transthoracic ultrasound was not useful, TEE, CT and MRI had a diagnostic ability for IMH, with sensitivities of 100% each [8]. Different diagnosis is essential because both, the initial and the final management may be completely different.

Natural history and predictors for progress and complications of IMH.

There is some controversy concerning the natural history of acute IMH. It is known that IMH may either progress or regress in an extend way. [4,23,38,39]. It originates from ruptured vasa vasorum in medial wall layers and results in an aortic wall infarct that may bring about a secondary tear, causing finally in some cases, a classic aortic dissection [23,24]. IMH should be affronted with attention; in fact, aortic IMH is considered as a precursor or a possible cause of a later dissection [6,6,20]. Whereas IMH resorption has been reported in only 10% of cases, never has resorption of aortic dissection been reported [7,8,40,41]. Studies of Kaji S et al and Neri E et al in 1999 and then Kaji S et al again in 2002 have suggested that IMHs reflect a more benign condition in which aggressive medical therapy and serial imaging may allow watchful waiting and the avoidance of surgery in some patients [42-44]. In IRAD study which registered 1010 patients with acute aortic dissection, 58 (5.7%) of them had IMH [27]. They showed an association between increasing hospital mortality and the proximity of IMH to the aortic valve, regardless of any medical or surgical treatment [2,27]. According to the international bibliography, [8,45,46], the IMH evolves to 1) resumption, 2) progression to classic aortic dissection, or 3) formation of an aneurysm within 30 days of hospital admission. A rate of 9 out of 12 deaths with IMH occurred in the ascending aorta, has been reported [2,27]. The presence of IMH in the ascending aorta is commonly considered as an independent factor of progression to aortic aneurysm formation, aortic rupture and/or dissection [45,46]. However, type A or proximal IMH is no longer related to early death when surgical intervention is performed [8,45,46]. According to Neinaber et al [8], a closer look reveals that 75% of proximal IMH patients died or had surgical replacement by the time of follow up. Alternatively, IMH of the descending aorta (type B or distal), may be treated conservatively or through endovascular intervention as elective cases [8,24,46,47]. According to a study [28] on morbidity and mortality for 168 patients with IMH, in 25% of ascending aortic IMH and in 13% of descending IMH led to aortic dissection, in 28% and 9% to aortic rupture, in 28% and 76% to stabilisation, respectively. In this study the 30-day mortality was 18% with surgical repair of proximal IMH, and 33% with surgery to distal IMH compared to 60% and 8% with medical treatment of type A and type B IMH, respectively [28]. Considering a 12% early mortality after surgery, and a 24% death rate with medical treatment, global experience from the International Registry of Aortic Dissection determined a tendency for a better outcome after surgery of proximal IMH [8,46,47]. It seems that IMH is similar to aortic dissection or better, to chronic aortic dissection. According to another study, the high risk of "wait and see" in type A IMH, is 55% early mortality with conservative - medical treatment compared to 8% with surgical repair [48]. However, in 10 out of 22 patients (45%) with type A IMH underwent surgical repair and four cases after medical management developed cardiac tamponade [49]. In another report [47] tamponade was observed in two out of three patients with type A IMH surviving medical treatment. According to records,, age and the use of β-blockers constitute factors of determining the progression of the IMH. In a report, only 7% of IMH with late progression were treated with β-blockers compared to 49% of IMH patients without late progression patients [50]. In a study, predictor of late progression of IMH is the younger age (<49 years) [8], and medication without β-blockers [8,45]. However, analysis of IMH confirmed better long term outcome in patients treated with β-blockers [47]. β-blockers protect by reducing aortic wall stress and the systolic arterial blood pressure [28,50]. Several controversies are present in this issue. There are not fixed predictor factors for early or late progression of IMH. Large series are necessaries in this setting. The observation that older age (> 55 years) at initial diagnosis of IMH has a better long term prognosis may be explained by more focal microscars along the aortic wall inherently limiting the longitudinal progression of IMH [8,20,22]. In reality, the IMH is considered [3] a class 2 aortic dissection.

## Medical or surgical management of IMH?

**Figure 3:**
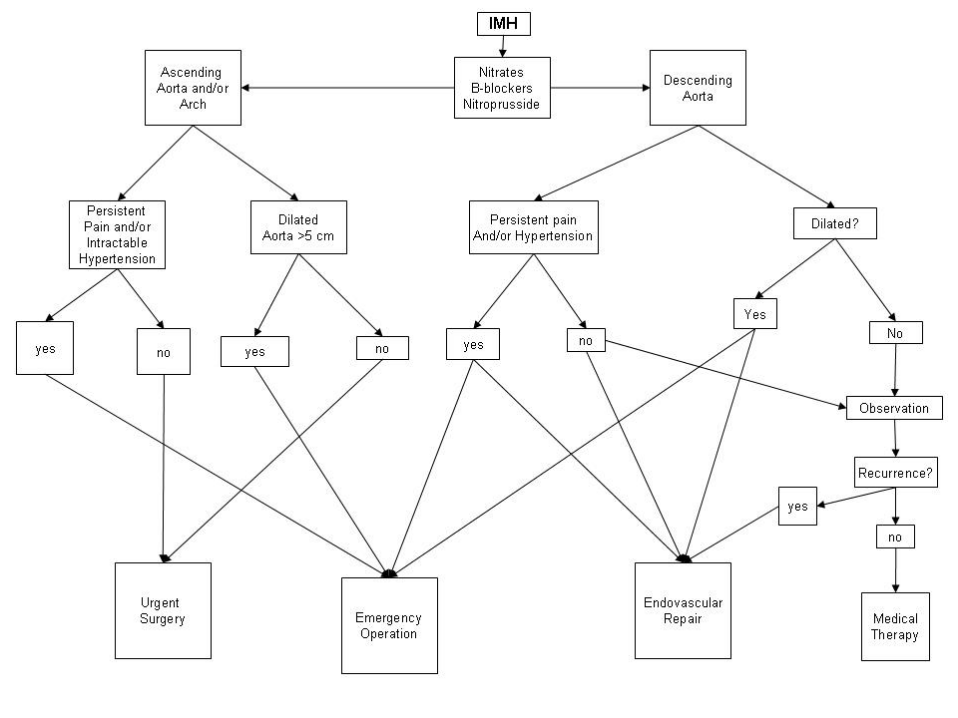
**The possible ways of treatment of the intramural haematoma of the thoracic aorta taking in consideration its location, the clinical presentation and the aortic diameter**.

Initial medical treatment, endovascular surgery or classic, open surgery is the common treatment of IMH. In the algorithm proposed we can see the different therapeutic strategies used in the treatment of IMH. We should bear in mind that IMH as "acute aortic syndrome" is indicative of a dynamic process and imminent events, so we should place our attention on detailed diagnostic confirmation with subsequent treatment by either surgical repair or interventional stent-graft placement [45,51]. Persistent and/or recurrent pain despite aggressive medical treatment, or repetitive pleural effusion, is an important indicator of disease progression [51] and represents a blatant indication for surgical or interventional handling [43,49]. If pericardial tamponade is diagnosed, pericardiocentesis as an initial therapeutic step before surgery may be dangerous because it reduces intrapericardial pressure and therefore may cause recurrent pericardial bleeding and sudden death [49]. Similar to type A and B aortic dissection, surgery is advisory at patients with type-A (ascending aorta) IMH and initial medical therapy at patients with type-B (descending aorta) IMH [35,44]. β-blockers protect by reducing aortic wall stress and the systolic arterial blood pressure [50]. However, in type B IMH surgical intervention is not a preferable way of treatment. We can perform a surgery in case of persistent pain, dilated (more than 5 cm), descending aorta and in elective patients. Alternatively, stent-grafting could be a perfect treatment especially if co morbidities are present. According to records, we are convinced that for patients with type A IMH, the classic open intervention is the correct way of treatment. Axillary cannulation for the extracorporeal circulation connection is usually performed with optimal brain and visceral perfusion [35]. Nonetheless, most cardiologists, as well as cardiac surgeons stand share the opinion that acute IMH involving the ascending aorta should be managed surgically because of an unacceptably high mortality rate following this medical treatment [4,7,8,23,24]. We take sides with this view as we have illustrated in our algorithm (figure 3). Unlike classic aortic dissection, IMH has no mechanisms of decompression by a re-entry tear [29,39,47,49]. According to the author's opinion, the ideal treatment for patients with IMH may be as the algorithm in the figure 3. We describe in this setting the ways of handling, considering the location, the symptoms and the aortic diameter.

## Conclusion

IMH is a rare but potentially lethal disease of the aorta. Nevertheless, pathogenesis and risk factors should be examined, in detail. Clinical manifestations, diagnosis, and management of acute aortic syndrome should be codified for rapid and accurate treatment. IMH of the aorta is a potentially lethal disorder with frequent conclusion to aortic rupture, dissection or aneurysm. Short term prognosis is extremely serious in IMH involving the ascending aorta, and surgical repair improves the outcome. IMH of the descending aorta, especially when confined to a short segment or without dilatation has a better outcome. Endovascular treatment is an alternative way of treatment in individual cases with acceptable results. Long term prognosis, may be more beneficial from chronic effective β blockers regardless of surgical repair.

## Competing interests

The authors declare that they have no competing interests.

## Authors' contributions

All authors: 1. have made substantial contributions to conception and design, or acquisition of data, or analysis and interpretation of data; 2. have been involved in drafting the manuscript or revisiting it critically for important intellectual content; 3. have given final approval of the version to be published.
